# Synthesis and evaluation of smart drugs with integrated functions for identifying and treating oxidative microenvironments associated with cellular ferroptosis

**DOI:** 10.1002/smo.20240048

**Published:** 2024-10-21

**Authors:** Yibo Zhang, Rui Cai, Yu Ding, Jiangye Zhang, Changxu Ning, Jiangcheng Zeng, Zhongxiang Zhou, Shisheng Wang, Yueqing Li, Xiuhan Guo

**Affiliations:** ^1^ Department of Pharmaceutical Engineering School of Chemical Engineering State Key Laboratory of Fine Chemicals Dalian University of Technology Dalian Liaoning China; ^2^ Instrumental Analysis Center Dalian University of Technology Dalian Liaoning China; ^3^ Department of Pharmacy Dalian Rehabilitation Recuperation Center Dalian China; ^4^ Ningbo Institute of Dalian University of Technology Ningbo Zhejiang China

**Keywords:** ferroptosis inhibitor, oxidative microenvironment, recognizing, space‐time controlled release

## Abstract

Ferroptosis is a novel form of cell death driven by oxidative damage, and is implicated in various pathological conditions, including neurodegenerative diseases, retinal damage, and ischemia‐reperfusion injury of organs. Inhibiting ferroptosis has shown great promise as a therapeutic strategy for these diseases, underscoring the urgent need to develop effective ferroptosis inhibitors. Although Ferrostatin‐1 (Fer‐1) is a potent ferroptosis inhibitor, its susceptibility to oxidation and metabolic inactivation limits its clinical utility. In this study, the accumulation of peroxides and the resulting oxidative damage in the cellular microenvironment during ferroptosis were utilized to design Ferrostatin‐1 prodrugs with reactive oxygen species‐responsive features. This approach led to the development of a series of ferroptosis inhibitors that were capable of recognizing oxidative damage in diseased areas, allowing for targeted release and improved stability. The novel compounds demonstrated significant inhibitory effects and selectivity against RSL‐3‐induced ferroptosis in HK‐2 cells, with compound a1 exhibiting an EC50 of 15.4 ± 0.7 μM, outperforming Fer‐1. These compounds effectively identify the oxidative microenvironment associated with ferroptosis, enabling the targeted release of Fer‐1, which prevents lipid peroxide accumulation and inhibits ferroptosis. This strategy holds promise for treating diseases related to ferroptosis, offering a targeted and intelligent therapeutic approach.

## Graphical abstract

In our work, a novel series of a‐compounds was successfully designed and synthesized based on the structure of fer‐1. These novel compounds not only enhanced the stability of fer‐1 but also demonstrated intelligent responsiveness to the intracellular ferroptosis microenvironment. This allowed for spatiotemporal‐specific release, a characteristic not seen in existing ferroptosis inhibitors. Among the a‐series compounds, compound a1 exhibited particularly strong activity, and low toxicity in normal cells, highlighting its selectivity as a ferroptosis inhibitor.

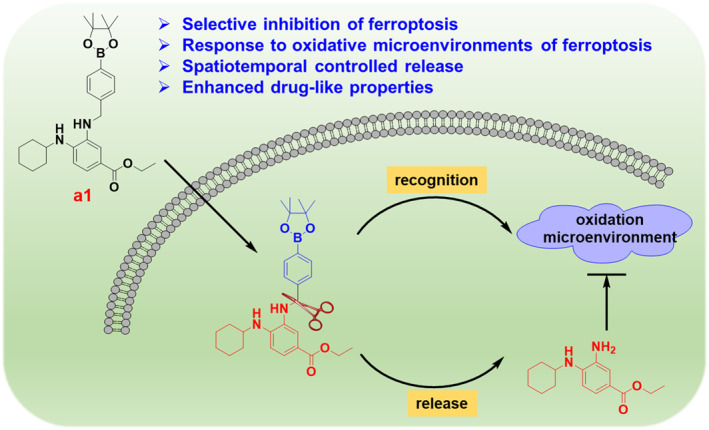

## 1 INTRODUCTION

Ferroptosis is a regulated form of cell death characterized by the involvement of iron ions, elevated levels of reactive oxygen species (ROS) such as H_2_O_2_ and hypochlorous acid, and the accumulation of lipid peroxides that ultimately lead to cell death.[^1^, ^2^, ^3^, ^4^] This process is widely observed in various physiological and pathological conditions in the human body. On one hand, ferroptosis plays a role in natural processes, including neural development, cellular aging, immune response, and the clearance of cancer cells.[^4^, ^5^, ^6^] On the other hand, it is associated with the pathogenesis of numerous diseases, such as traumatic brain injury,[^7^, ^8^] neurodegenerative disorders,[^9^, ^10^, ^11^] acute liver and kidney injuries,[^12^, ^13^, ^14^] and ischemia‐reperfusion injury (IRI).[^15^, ^16^, ^17^] The involvement of ferroptosis in these conditions suggests significant potential for therapeutic intervention by inhibiting this form of cell death.[^6^, ^18^, ^19^] For example, research by Li et al. demonstrated that dexmedetomidine can reduce liver IRI by inhibiting ferroptosis.^[20]^ Zhang et al. found that ischemic brain injury in neonatal rats involves ferroptosis and successfully mitigated this damage using the ferroptosis inhibitor lip‐1.^[21]^ These findings underscore the potential of developing ferroptosis inhibitors as a promising approach for treating diseases that currently lack effective clinical treatments.

**FIGURE 1:**
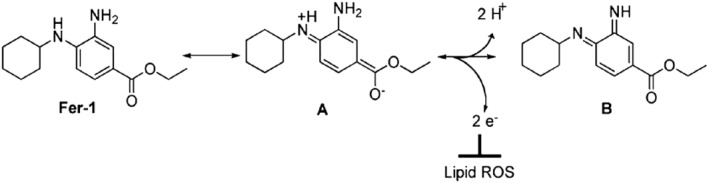
Mechanism of action of fer‐1.

Lipophilic antioxidants are currently the most extensively studied class of ferroptosis inhibitors, including ferrostatin‐1 (Fer‐1),[^1^, ^22^] liproxstatin‐1 (Lip‐1),^[23]^ phenoxazine (PNX) derivatives,^[24]^ and vitamin K.^[25]^ Among these, Fer‐1, as the first ferroptosis‐specific inhibitor to be systematically studied, has demonstrated strong anti‐ferroptotic activity. It is widely used as a standard reference compound to evaluate the effectiveness of other ferroptosis inhibitors in research. Its mechanism^[22]^ of action is illustrated in Figure 1.

Although Fer‐1 exhibits high ferroptosis inhibitory activity and specificity, its clinical application is significantly hindered due to its susceptibility to oxidation and short half‐life.[^26^, ^27^] Researchers have explored various modifications to enhance the stability and efficacy of Fer‐1, focusing mainly on replacing ester bonds or altering its core structure, as seen in compounds like UAMC‐2418, UAMC‐3203, and 2‐amino‐6‐methyl‐phenol derivatives.[^28^, ^29^, ^30^, ^31^, ^32^]

Phenylboronic esters and phenylboronic acid groups are commonly used ROS‐responsive moieties that can protect amino or hydroxyl groups and release the drug in response to elevated H_2_O_2_ levels.[^33^, ^34^] In this study, benzyl boronate/boronic acid groups were used to mask the aryl primary amino groups in fer‐1 which are prone to oxidation. This approach leverages the elevated levels of hydrogen peroxide (H_2_O_2_) characteristic of the ferroptosis microenvironment to create prodrugs (a1–a5) that can respond to the ferroptosis microenvironment and release the drug accordingly. The influence of different linkage methods on drug activity was evaluated. In addition, the toxicity, release profile, specificity of ferroptosis inhibition, and effects on ROS levels in ferroptotic cells were assessed for these compounds.

## 2 RESULTS AND DISCUSSION

### 2.1 Chemistry

**FIGURE 2:**
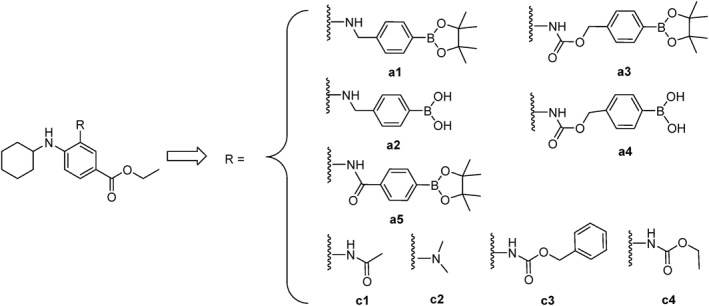
Structures of designed compounds.

In this study, a series of Fer‐1 prodrug molecules (**a1**–**a4**) were designed. These prodrugs utilized two different linkage structures to connect boronic acid and boronate ester groups. Both boronic acid and boronate ester groups have been demonstrated to be responsive to oxidative environments, enabling the release of the amino group moiety (Figure 2).

In this work, ethyl 4‐chloro‐3‐nitrobenzoate (**1**) was heated with cyclohexylamine in the presence of potassium carbonate (K_2_CO_3_) in DMSO to produce compound **2**. The nitro group of compound **2** was then reduced under a hydrogen atmosphere using palladium on carbon to yield 3‐amino‐4‐(cyclohexylamino) benzoate (**3**), also known as ferrostatin‐1 (fer‐1).

4‐(Hydroxymethyl)phenylboronic acid pinacol ester (**4**) reacted with triphosgene in toluene in the presence of sodium carbonate (Na_2_CO_3_) to form compound **5**. Compound **3** underwent a substitution reaction with either 4‐bromomethylphenylboronic acid pinacol ester or compound **5** under basic conditions to produce compound **a1** or **a3**, respectively. Additionally, compound **3** reacted with 4‐(tetramethyl‐1,3,2‐dioxaborolan‐2‐yl) benzoic acid in DCM in the presence of HOBt, EDCI, and DIPEA to form compound **a5**. Compounds **a1** or **a3** were hydrolyzed in DMF with HCl to obtain compounds **a2** or **a4**. All target compounds (**a1**–**a5**) were novel and had not been reported previously. Their chemical structures were confirmed using ^1^H‐NMR and mass spectrometry (see Supporting Information S1 for details of synthetic route).

To validate the design strategy, control compounds (**c1**–**c4**) were also synthesized. These compounds were produced by reacting compound **3** with the corresponding acyl chlorides or methyl iodide in the presence of DIPEA in THF. The chemical structures of **c1**–**c4** were confirmed using ^1^H‐NMR and mass spectrometry (Figure 2).

### 2.2 Cytotoxicity

Human renal proximal tubular epithelial cells (HK‐2) were incubated with various concentrations of the synthesized compounds, and cell viability was assessed using the MTT assay. The results showed that all the designed compounds exhibited low cytotoxicity with lower toxicity than fer‐1 at a concentration of 100 μM (see Supporting Information S1). These findings suggest the potential of these compounds for further activity evaluation.

### 2.3 In vitro inhibitory activity against ferroptosis and structure‐activity relationships

**FIGURE 3:**
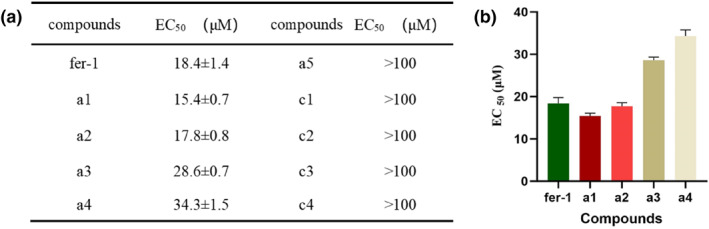
(a) Ferroptosis inhibitory activity of compounds in vitro; (b) EC50 values analysis of active compounds. *p* < 0.01 compared with control group.

Ferroptosis in human renal proximal tubular epithelial cells (HK‐2) was induced using 4 μM RSL‐3, and the activities of the compounds were characterized by their EC_50_ values. As shown in Figure 3, compounds **a1**–**a4** demonstrated significant ferroptosis inhibition, with compound a1 showing the highest activity (EC_50_ = 15.4 ± 0.7 μM), slightly surpassing that of fer‐1. In contrast, compounds **a5** and **c1**–**c4** did not exhibit ferroptosis inhibitory activity (EC_50_ > 100 μM). The lack of ferroptosis inhibition by **a5**, despite its similar boronate ester structure to **a1**–**a4**, may be attributed to the presence of an amide bond that restricts the release of fer‐1, even though the boronate ester recognized the oxidative environment. Compounds **c3** and **c4**, which contain carbamate groups reported to be cleavable by ROS, also failed to show activity. These results indicate that boronic acid and boronate ester groups play a crucial role in responding to oxidative environments. Additionally, the lack of activity in compounds **c1** and **c2** suggests that the release of primary amines is essential for achieving ferroptosis inhibition.

Moreover, compound **a2** exhibited lower activity than **a1**, and **a4** was less active than **a3**, indicating that boronate esters are more effective than boronic acids. Overall, the above analysis shows that such compounds have the ability to respond to ferroptosis oxidation microenvironment, exert ferroptosis inhibitory activity, and can effectively protect amino groups to avoid oxidation deactivation. Compound **a1**, which has the strongest the strongest anti‐ferroptosis activity, was used for subsequent activity tests.

### 2.4 Specificity of inhibiting cell death

**FIGURE 4:**
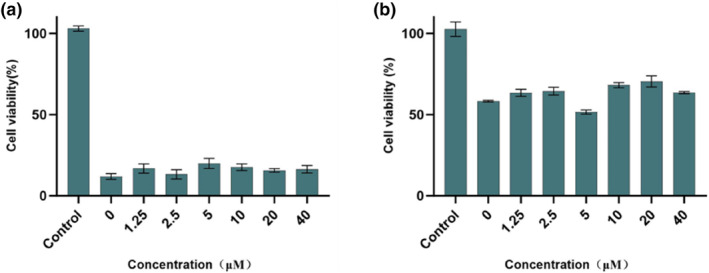
(a) Effect of **a1** on apoptosis; (b) Effect of **a1** on necrosis. *p* < 0.01 compared with control group.

Camptothecin (CPT) is known as an effective inducer of apoptosis. In this work, HK‐2 cells were treated with 20 μM CPT to induce apoptosis. To evaluate the effect of compound **a1** on cell survival, HK‐2 cells were co‐incubated with varying concentrations of compound a1 along with CPT. Cell viability was assessed using the MTT assay, and results were compared with a control group, as shown in Figure 4a. The data indicated that CPT effectively induced apoptosis in HK‐2 cells. However, the addition of different concentrations of compound **a1** did not prevent cell death. These findings suggest that compound **a1** is unable to inhibit apoptosis under the tested conditions.

In addition, a necrosis model in HK‐2 cells was established using 5 μg/mL cisplatin. To investigate the potential protective effects of compound **a1**, the cells were co‐incubated with various concentrations of compound a1 alongside cisplatin treatment. Cell viability was measured using the MTT assay, and the results were compared with those of the model group, as shown in Figure 4b. The findings demonstrated that cisplatin significantly reduced cell viability. However, the presence of compound **a1** did not reverse this decrease in viability. These results indicated that compound a1 was ineffective in inhibiting necrosis under the tested conditions.

The results were compared to evaluate the inhibitory effects of compound **a1** on ferroptosis, apoptosis, and necrosis. The results indicated that compound **a1** selectively protected against ferroptosis in HK‐2 cells but did not demonstrate a significant protective effect against apoptosis or necrosis.

### 2.5 Drug release in solution in a cellular oxidative environment that simulates ferroptosis

Ferroptosis is a form of cell death that depends on iron ions and is caused by oxidative damage due to the accumulation of lipid peroxides. H_2_O_2_ was used to replicate the oxidative microenvironment characteristic of ferroptosis. Compound **a1** was dissolved in a PBS: DMSO = 9:1 solution, and varying amounts of H_2_O_2_ were added to the **a1** solution to simulate the release of the compound in a ROS‐induced environment in vitro.

As shown in Figure 4, the addition of an equimolar amount of H_2_O_2_ to the solution containing compound **a1** resulted in the appearance of a [fer‐1+H]^+^ peak at 263.04 in the mass spectrum. Meanwhile, two new peaks at 369.07 and 391.06 were observed, which are presumed to correspond to the intermediate product **a’**, identified as [a’+H]^+^ and [a’+Na]^+^ peaks, respectively. At the same time, the [a1+H]^+^ peak at 479.24 and the [a1+Na]^+^ peak at 501.24 were also detected.

**FIGURE 5:**
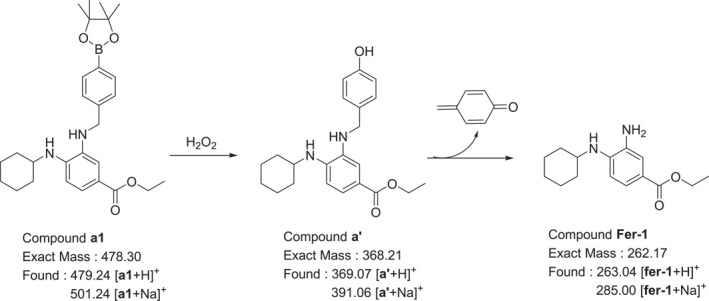
Analysis of fer‐1 release from compound **a1** in a simulated oxidizing environment.

When **5** equivalents of H_2_O_2_ were added, compound **a1** was nearly completely reacted as evidenced by the absence of its corresponding ion peaks in the mass spectrum. Only a clear signal of fer‐1 was detected, with minimal presence of the intermediate product **a’**, indicating that under these conditions, **a1** was almost entirely converted to fer‐1. These results demonstrate that compound **a1** can effectively release the active drug fer‐1 in a simulated in vitro oxidative environment (Figure 5).

### 2.6 Determination of intracellular total ROS level

**FIGURE 6:**
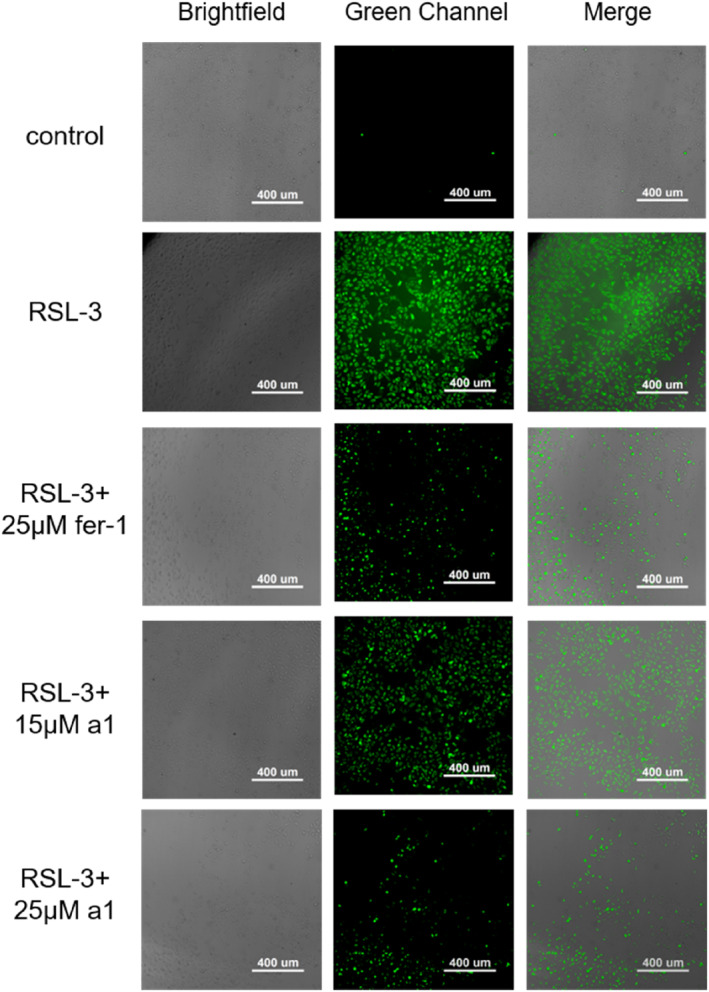
The impact of compound a1 on the intracellular reactive oxygen species levels in ferroptotic cells.

To further evaluate the protective mechanism of compound a1 against ferroptosis, its effect on intracellular ROS levels was assessed. The DCFH‐DA dye, in combination with a high‐content imaging system, was used to visually observe intracellular ROS levels through fluorescence. RSL‐3, an effective inducer of ferroptosis, was used to induce ferroptosis in HK‐2 cells. As shown in Figure 6, the ROS levels in the RSL‐3‐induced group increased significantly. By contrast, the addition of fer‐1 or compound **a1** led to a noticeable reduction in ROS levels. At the same concentration of 25 μM, compound **a1** resulted in lower ROS levels compared to fer‐1, indicating that compound **a1** is more effective at scavenging ROS than fer‐1. A positive correlation was observed between the reduction in ROS levels and increasing concentrations of compound **a1**.

### 2.7 Determination of intracellular lipid reactive oxygen species

**FIGURE 7:**
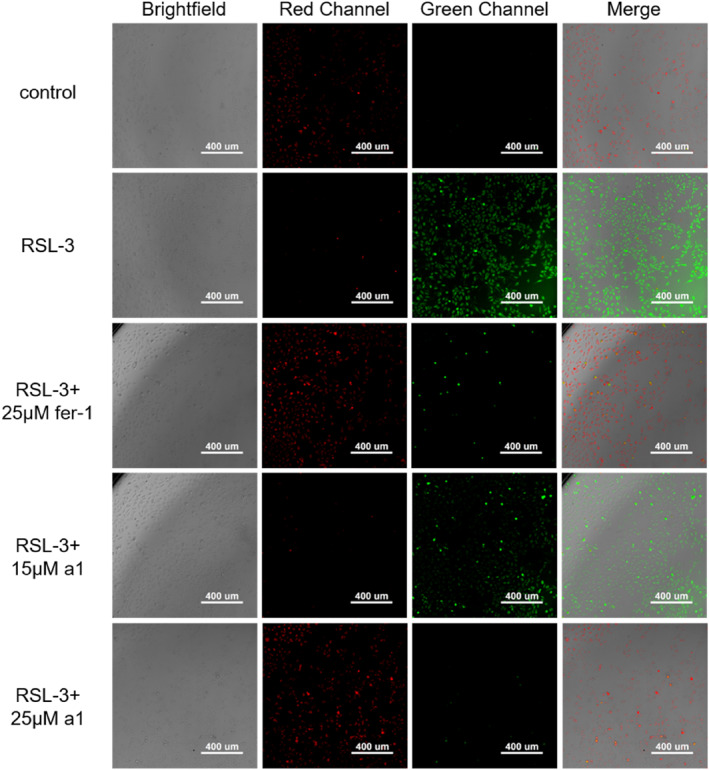
Effect of compound **a1** on lipid reactive oxygen species levels within ferroptotic cells.

C11‐BODIPY dye was utilized alongside a high‐content imaging system to visually observe the intracellular levels of ROS through fluorescence imaging. Lipid ROS is a key marker of ferroptosis. As shown in Figure 7, a significant increase in green fluorescence was detected in the RSL‐3‐treated group, indicating elevated levels of lipid ROS. This suggests that the addition of RSL‐3 induces ferroptosis by increasing intracellular lipid ROS levels. Conversely, treatment with either fer‐1 or compound **a1** resulted in a reduction of lipid ROS as evidenced by decreased green fluorescence and more pronounced red fluorescence from the reduced form of the probe. In experiments using compound **a1**, the concentration of lipid ROS was found to be lower compared to the control substance fer‐1. When cells were treated with 25 μM of **a1**, no significant green fluorescence was observed, indicating that compound a1 had a superior ability to scavenge lipid ROS compared to fer‐1. This result was consistent with cell viability evaluations. Furthermore, a comparison across different concentrations of compound **a1** showed that the reduction in lipid ROS levels was positively correlated with the concentration of **a1**. These findings suggest that compound a1 effectively performed similar functions to fer‐1 by reducing intracellular ROS and lipid ROS concentrations, inhibiting ferroptosis, and protecting the cells.

### 2.8 ADMET analysis

To further investigate the potential drug properties of the synthesized compounds, pharmacokinetic predictions were performed using the ADMET Descriptors module in Discovery Studio software. The results of these predictions are presented in Table 1. Except for compound **a3**, all compounds demonstrated solubility within an acceptable range. The compounds also exhibited the ability to penetrate the Blood‐brain barrier (BBB), suggesting their potential use in the evaluation of therapeutic activity for neurological diseases related to ferroptosis. In addition, the compounds were shown to be metabolized normally and did not inhibit the CYP2D6 enzyme. No significant hepatotoxicity was observed, and all compounds were capable of intestinal absorption and binding to plasma proteins. Further detailed pharmacokinetic studies, both in vitro and in vivo, may be required for confirmation.

**TABLE 1:** Prediction results of ADMET.

Comps.	Solubility level^a^	BBB penetration level^b^	CYP2D6 binding	Hepato‐toxicity	Absorption level^c^	Plasma protein binding
a1	2	2	FALSE	FALSE	1	TRUE
a2	3	2	FALSE	FALSE	2	TRUE
a3	1	2	FALSE	FALSE	1	TRUE
a4	2	2	FALSE	FALSE	2	TRUE
Fer‐1	2	1	FALSE	FALSE	1	FALSE

*Note*: FALSE: no interaction; TRUE: interaction.

^a^ Solubility levels of the compounds: 0,1—the compound is insoluble or poorly soluble in water, 2 ∼ 4—soluble in water, 5—extremely soluble and difficult to make medicines.

^b^ Blood‐brain barrier (BBB) penetration level: The ability to penetrate the BBB increases from 0 to 3.

^c^ The level of absorption of a compound represents how easily the drug is absorbed through the intestine; A scale of 0–3 represents how easily the compound is absorbed by the intestine. 0 means easy to be absorbed by the intestine, 3 means extremely difficult to be absorbed by the intestine.

## 3 CONCLUSION

Ferroptosis, a novel form of cell death resulting from lipid peroxidation, was identified. The inhibition of ferroptosis is considered promising for the treatment of various diseases. This study addressed the structural instability issues associated with the ferroptosis inhibitor fer‐1. In response to the oxidative microenvironment present during ferroptosis, a novel series of a‐compounds was successfully designed and synthesized based on the structure of fer‐1. These compounds not only enhanced the stability of fer‐1 but also demonstrated intelligent responsiveness to the intracellular ferroptosis microenvironment. This allowed for spatiotemporal‐specific release, a characteristic not seen in existing ferroptosis inhibitors. Among the a‐series compounds, compound a1 exhibited particularly strong activity. In in vitro ferroptosis models, a1 had an EC50 of 15.4 μM, which was superior to that of fer‐1, with an EC50 of 18.3 μM, indicating its greater efficacy in inhibiting ferroptosis. Moreover, **a1** exhibited low toxicity in normal cells, highlighting its selectivity as a ferroptosis inhibitor.

The introduction of a ROS‐responsive mechanism in the a‐series compounds allowed for specific activation in ferroptosis‐related diseases while minimizing nonspecific distribution in healthy tissues, thereby reducing potential side effects. This design strategy not only enhanced the targeting ability of the compounds but also offered new possibilities for clinical applications, particularly in therapeutic scenarios requiring precise spatiotemporal control.

In conclusion, this study provides valuable tools for the treatment of ferroptosis‐related diseases with the aim of advancing these intelligent ferroptosis inhibitors toward clinical application, ultimately offering more effective therapeutic options for patients.

## CONFLICT OF INTEREST STATEMENT

The authors declare no conflicts of interest.

## ETHICS STATEMENT

No animal or human experiments were involved in this study.

## Supporting information

Supporting Information S1

## Data Availability

The data that support the findings of this study are available in the supplementary material of this article.
