# Supplementary material for: Synthesis and evaluation of smart drugs with integrated functions for identifying and treating oxidative microenvironments associated with cellular ferroptosis
Source: Smart Mol. 2024 Oct 21;3(2):e20240048. doi: 10.1002/smo.20240048 (PMC12262006; doi:10.1002/smo.20240048)
Supplement: Supplementary file 1 — Supporting Information S1 [file SMO2-3-e20240048-s001.docx]

**Supporting Information**

Synthesis and Evaluation of Smart Drugs with Integrated Functions for Identifying and Treating Oxidative Microenvironments Associated with Cellular Ferroptosis

Yibo Zhang^a^, Rui Cai^b^, Yu Ding^d^, Jiangye Zhang^a^, Changxu Ning^a^, Jiangcheng Zeng^a^, Zhongxiang Zhou^a^, Shisheng Wang^a.c^*, Yueqing Li^a,c^*，Xiuhan Guo^a,c^*

^a^State Key Laboratory of Fine Chemicals, Department of Pharmaceutical Engineering, School of Chemical Engineering, Dalian University of Technology, Dalian 116024, Liaoning, China

^b^Instrumental Analysis Center, Dalian University of Technology, Dalian 116024, Liaoning, China

^c^Ningbo Institute of Dalian University of Technology, Ningbo 315016, Zhejiang, China

^d^Department of Pharmacy, Dalian Rehabilitation Recuperation Center, Dalian 116013, China

**4. Experimental section**

**4.1. Chemistry**

Unless otherwise stated, all commercial reagents obtained from Aladdin, Energy Chemical and Xilong Chemical were used without purification. 200-300 mesh silica gel was used for column chromatography. Using CDCl_3_ or DMSO-d_6_ as the deuterated solvent, ^1^H and ^13^C NMR spectra were recorded on Bruker Avance II 400 M, Varian DLG 400, Bruker Avance NEO 600 M NMR Spectroscopy instruments. The NMR data was analyzed using MestReNova software. High-resolution mass spectra were recorded on the LC/Q-Tof MS mass spectrometer.

Figture S1. Synthetic route of compounds **a1**~**a5**

Figture S2. Synthetic route of compounds **c1**~**c4**

**4.1.1. Synthesis of compound 2**

Compound **1** (7.0105 g, 30.5 mmol) was dissolved in 30mL of DMSO to obtain a light yellow solution. Cyclohexylamine (3.9361 g, 39.7 mmol) and K_2_CO_3_ (8.4280 g, 61.1 mmol) were added to the bottle, stirred at 60℃ overnight, and the solution turned orange-red. The reaction solution was diluted with 40mL of ethyl acetate, washed three times, washed once with saturated saline water, and the organic phase was concentrated to give 6.595 g of orange-red solid, with a crude product yield of 74%. The compound can be used directly in the next step without purification.

**4.1.2. Synthesis of compound 3**

Compound **2** was dissolved in 30 mL of ethanol, 50 mg of Pd/C was added, stirred at room temperature overnight under hydrogen atmosphere, and the Pd/C was removed by filtration with celite. The ethanol was removed by rotary evaporation to obtain a brown-black solid. The crude was purified by silica gel column chromatography with PE/EtOAc (5:1) as an eluent to obtain compound **3**, which was crystallized in white to light pink grains. The yield was 91%. ^1^H NMR (600 MHz, Methanol-*d*_4_) *δ* 7.42 (dd, *J* = 8.4, 2.0 Hz, 1H), 7.36 (d, *J* = 2.0 Hz, 1H), 6.56 (d, *J* = 8.4 Hz, 1H), 4.25 (q, *J* = 7.2 Hz, 2H), 3.34 (m, 1H), 2.06 (m, 2H), 1.79 (m, 2H), 1.68 (m, 1H), 1.42 (m, 2H), 1.33 (t, *J* = 7.2 Hz, 3H), 1.25 (m, 3H). ESI-MS calculated for C_15_H_23_N_2_O_2:_ 263.17; found: 263.17 [M+H]^+^.

**4.1.3. Synthesis of compound a1**

Fer-1 (0.2012 g, 0.76 mmol) was dissolved in 5.0 mL of anhydrous DMF, 4-bromomethylphenylboronic acid pinacol ester (0.2280 g, 0.76 mmol), K_2_CO_3_ (0.1612 g, 1.52 mmol) were added, stirred at 60℃ for 3 hours, and the reaction stopped as monitored by TLC. Added 15mL of ethyl acetate, washed twice, wash once with saturated sodium chloride solution, and dry the organic phase over anhydrous Na_2_SO_4_. Rotary evaporation gave the crude product, and column chromatography on petroleum ether: ethyl acetate =9:1 gave compound **a1** as a light yellow solid. The yield was 74%. ^1^H NMR (400 MHz, DMSO-*d*_6_) *δ* 7.64 (d, *J* = 7.7 Hz, 2H), 7.37 (d, *J* = 7.7 Hz, 2H), 7.22 (dd, *J* = 8.4, 2.0 Hz, 1H), 6.88 (d, *J* = 2.0 Hz, 1H), 6.49 (d, *J* = 8.4 Hz, 1H), 5.56 (t, *J* = 5.5 Hz, 1H), 5.14 (d, *J* = 7.2 Hz, 1H), 4.34 (d, *J* = 5.5 Hz, 2H), 4.12 (q, *J* = 7.2 Hz, 2H), 3.35 (m, 1H), 1.99 (m, 2H), 1.75 (m, 2H), 1.62 (m, 1H), 1.41–1.32 (m, 2H), 1.28 (s, 12H), 1.25 (m, 1H), 1.23 (m, 2H), 1.20 (t, *J* = 7.1 Hz, 3H). ESI-MS calculated for C_28_H_40_BN_2_O_4_: 478.30; found: 479.24 [M+H]^+^ .

**4.1.4. Synthesis of compound a2**

Compound **a1** (0.0508 g, 0.11 mmol) was dissolved in 5mL of 1M hydrochloric acid and stirred for 0.5 h. Added 10mL of ethyl acetate for extraction, and dry the organic phase once with an equal volume of saturated sodium chloride solution. The organic layer was concentrated by rotary evaporation to about 2mL. Added 5mL of hexane, and a yellow solid precipitate appears. After sufficient precipitation, filter and wash to obtain a yellow solid, compound **a2**. The yield was 94%. ^1^H NMR (400 MHz, DMSO-d_6_) *δ* 8.46 (s, 2H), 7.66 (d, *J* = 7.6 Hz, 2H), 7.40 (d, *J* = 7.6 Hz, 2H), 7.25 (dd, *J* = 8.5, 1.9 Hz, 1H), 6.88 (d, *J* = 1.9 Hz, 1H), 6.50 (d, *J* = 8.5 Hz, 1H), 5.56 (t, *J* = 5.5 Hz, 1H), 5.14 (d, *J* = 7.2 Hz, 1H), 4.36 (d, *J* = 5.5 Hz, 2H), 4.13 (q, *J* = 7.2 Hz, 2H), 3.36 (m, 1H), 2.00 (m, 2H), 1.80 (m, 2H), 1.66 (m, 1H), 1.36 (m, 2H), 1.27 (m, 1H), 1.24 (m, 2H), 1.21 (t, *J* = 7.1 Hz, 3H). ESI-MS calculated for C_22_H_28_BN_2_O_4_: 395.22; found: 395.18 [M-H]^-^.

**4.1.5. Synthesis of compound 5**

4-Hydroxymethylphenylboronic acid pinacol ester (0.1002 g, 0.43 mmol) was dissolved in 10mL of toluene, added bis(trichloromethyl) carbonate (0.1884 g, 0.64 mmol), stirred to dissolve, added Na_2_CO_3_ (0.1823 g, 1.72 mmol), and stirred at room temperature for 1h. After the reaction, the solid was filtered off, the filtrate was extracted once with an equal volume of deionized water, the organic layer was washed once with saturated saline water, dried over anhydrous Na_2_SO_4_, and spun off to obtain Compound **5**, a colorless, transparent oily liquid. The yield was 96%. Not purified, used in the next reaction.

**4.1.6. Synthesis of compound a3**

To synthesize compound **a3**, fer-1 (0.2006 g, 0.76 mmol) was dissolved in 5.0 mL of anhydrous dichloromethane. Compound **5** (0.2270 g, 0.76 mmol) and triethylamine (0.154 g, 1.52 mmol) were then added to the solution. The mixture was stirred at room temperature for 12 hours, after which TLC confirmed the reaction had completed. The reaction mixture was diluted with 15 mL of ethyl acetate and washed twice with an equal volume of water, followed by one wash with an equal volume of saturated sodium chloride solution. The organic phase was dried over anhydrous Na_2_SO_4_. After rotary evaporation to concentrate the solution, the crude product was purified using column chromatography with a petroleum ether acetate ratio of 7:1, yielding compound **a3** as a white solid with a 54% yield. ^1^H NMR (400 MHz, DMSO-d6) *δ* 9.11 (s, 1H), 8.46 (d, *J* = 2.2 Hz, 1H), 7.76 (dd, *J* = 9.0, 2.2 Hz, 1H), 7.73 – 7.66 (m, 2H), 7.34 – 7.26 (m, 2H), 7.19 (d, *J* = 9.0 Hz, 1H), 5.82 (d, *J* = 7.9 Hz, 1H), 5.17 (t, *J* = 1.0 Hz, 2H), 4.32 (q, *J* = 6.4 Hz, 2H), 3.48 (dq, *J* = 7.9, 4.7 Hz, 1H), 1.88 – 1.75 (m, 2H), 1.70 – 1.32 (m, 10H), 1.23 (s, 13H). ESI-MS calculated for C_29_H_40_BN_2_O_6_: 523.29; found: 523.16 [M+H]^+^.

**4.1.7. Synthesis of compound a4**

To synthesize compound **a4**, **a3** (0.0506 g, 0.11 mmol) was added to a 25 mL round-bottom flask containing 5 mL of 1M hydrochloric acid. The mixture was stirred thoroughly for 30 minutes. Then, 10 mL of ethyl acetate was added for extraction. The organic layer was washed once with an equal volume of saturated sodium chloride solution and concentrated by rotary evaporation to approximately 2 mL. About 5 mL of n-hexane was added, resulting in the formation of a white solid precipitate. After allowing the precipitate to settle for about 1 hour, the solid was filtered and washed with a small amount of n-hexane, yielding compound **a4** as a white solid with a 92% yield. ^1^H NMR (400 MHz, DMSO-d6) *δ* 9.11 (s, 1H), 8.46 (d, *J* = 2.2 Hz, 1H), 8.28 (s, 2H), 7.76 (dd, *J* = 9.0, 2.2 Hz, 1H), 7.68 – 7.61 (m, 2H), 7.30 – 7.22 (m, 2H), 7.19 (d, *J* = 9.0 Hz, 1H), 5.82 (d, *J* = 7.9 Hz, 1H), 5.17 (t, *J* = 1.0 Hz, 2H), 4.32 (q, *J* = 6.4 Hz, 2H), 3.48 (dq, *J* = 7.9, 4.7 Hz, 1H), 1.88 – 1.75 (m, 2H), 1.72 – 1.32 (m, 11H). ESI-MS calculated for C_23_H_28_BN_2_O_6_: 439.21; found: 439.19 [M-H]^-^.

**4.1.8. Synthesis of compound a5**

To synthesize compound **a5**, fer-1 (0.1000 g, 0.38 mmol) was dissolved in 5.0 mL of anhydrous dichloromethane (DCM). Compound **8** (0.1141 g, 0.46 mmol) and HOBt (0.0622 g, 0.46 mmol) were added to the solution, and the mixture was stirred until homogeneous. EDCI (0.882 g, 0.46 mmol) and DIPEA (0.1970 g, 0.76 mmol) were then added, and the reaction was carried out under a nitrogen atmosphere with stirring at room temperature for 12 hours, monitored by TLC until completion. The reaction mixture was diluted with 10 mL of DCM and washed twice with an equal volume of water, followed by one wash with saturated sodium chloride solution. The organic layer was dried over anhydrous Na_2_SO_4_ and concentrated by rotary evaporation to yield the crude product. The crude product was purified using column chromatography with a petroleum ether acetate ratio of 20:1, yielding compound **a5** as a yellow-brown solid with a 37% yield. ^1^H NMR (400 MHz, DMSO-d6) *δ* 8.40 (d, *J* = 2.2 Hz, 1H), 7.83 (m, 2H), 7.78 (m, 2H), 7.75 (d, *J* = 2.2 Hz, 1H), 7.28 (d, *J* =8.0 Hz, 1H), 5.75 (d, *J* = 8.0 Hz, 1H), 4.32 (q, *J* = 6.4 Hz, 2H), 3.48 (m, 1H), 1.81 (m, 2H), 1.64 (m, 2H), 1.55 (m, 1H), 1.52 – 1.39 (m, 5H), 1.36 (t, *J* = 6.4 Hz, 3H), 1.23 (s, 12H). ESI-MS calculated for C_28_H_38_BN_2_O_5_: 493.30; found: 493.27 [M+H]^+^.

**4.1.9. Synthesis of compound c1**

To synthesize compound **c1**, fer-1 (0.1001 g, 0.38 mmol) was dissolved in 5.0 mL of anhydrous tetrahydrofuran (THF). Acetyl chloride (27 µL, 0.76 mmol) was then added, and the mixture was stirred using a magnetic stirrer until homogeneous. DIPEA (0.3940 g, 1.52 mmol) was added to the solution, and the reaction was carried out under a nitrogen atmosphere with stirring at room temperature for 12 hours, monitored by TLC until completion. After the reaction, 15 mL of ethyl acetate was added to dilute the mixture. The solution was washed twice with an equal volume of water, followed by a wash with saturated sodium chloride solution. The organic phase was dried over anhydrous Na_2_SO_4_ and concentrated using rotary evaporation to obtain the crude product. The crude product was purified by column chromatography using a petroleum ether acetate ratio of 15:1, yielding compound **c1** as a pale yellow solid with a 71% yield. ^1^H NMR (400 MHz, CDCl3) *δ* 7.82 (d, *J* = 8.6 Hz, 1H), 7.75 (s, 1H), 6.68 (d, *J* =8.6 Hz, 1H), 4.57 – 4.46 (m, 1H), 4.33 (d, *J* = 9.8 Hz, 2H), 3.35 (s, 1H), 2.22 (s, 3H), 2.04 (d, *J* = 12.1 Hz, 2H), 1.88 (s, 1H), 1.79 (s, 3H), 1.38 (s, 4H), 1.24 (d, *J* = 8.6 Hz, 3H). ESI-MS calculated for C_17_H_25_N_2_O_3_: 305.18; found: 305.19 [M+H]^+^.

**4.1.10. Synthesis of compound c2**

To synthesize compound **c2**, fer-1 (0.1002 g, 0.38 mmol) was dissolved in 5.0 mL of anhydrous tetrahydrofuran (THF). Methyl iodide (0.1082 g, 0.76 mmol) was then added, and the mixture was stirred using a magnetic stirrer until homogeneous. DIPEA (0.3940 g, 1.52 mmol) was added to the solution, and the reaction was carried out under a nitrogen atmosphere at room temperature for 10 hours. An additional amount of methyl iodide (0.1082 g, 0.76 mmol) was added, and the reaction continued for another 5 hours at room temperature, monitored by TLC until completion. The reaction mixture was then diluted with 15 mL of ethyl acetate. It was washed twice with an equal volume of water, followed by a wash with saturated sodium chloride solution. The organic layer was dried over anhydrous Na_2_SO_4_ and concentrated using rotary evaporation to yield the crude product. The crude product was purified by column chromatography using a petroleum ether acetate ratio of 20:1, resulting in compound **c2** as a yellow solid with an 85% yield. ^1^H NMR (400 MHz, DMSO-d6) δ 7.84 (dd, *J* = 9.2, 2.2 Hz, 1H), 7.52 (d, *J* = 2.2 Hz, 1H), 7.05 (d, *J* = 9.2 Hz, 1H), 5.24 (d, *J* = 8.0 Hz, 1H), 4.32 (q, *J* = 6.4 Hz, 2H), 3.49 (m, 1H), 1.88 – 1.75 (m, 2H), 2.93（s, 6H）1.65 (m, 2H), 1.56 (m, 1H), 1.46 (m, 5H), 1.36 (t, *J* = 6.4 Hz, 3H). ESI-MS calculated for C_17_H_27_N_2_O_2_: 291.20; found: 291.17 [M+H]^+^.

**4.1.11. Synthesis of compound c3**

To synthesize compound **c3**, fer-1 (0.1000 g, 0.38 mmol) was dissolved in 5.0 mL of anhydrous tetrahydrofuran (THF). Benzyl chloroformate (0.1301 g, 0.76 mmol) was added, and the mixture was stirred using a magnetic stirrer until homogeneous. DIPEA (0.3940 g, 1.52 mmol) was then introduced into the solution, and the reaction was carried out under a nitrogen atmosphere at room temperature for 15 hours, monitored by TLC until completion. The reaction mixture was diluted with 15 mL of ethyl acetate and washed twice with an equal volume of water, followed by a wash with saturated sodium chloride solution. The organic layer was dried over anhydrous Na_2_SO_4_ and concentrated by rotary evaporation to obtain the crude product. The crude product was purified using column chromatography with a petroleum ether acetate ratio of 15:1, resulting in compound **c3** as a white solid with a yield of 79%. ^1^H NMR (400 MHz, DMSO-d6) *δ* 9.11 (s, 1H), 8.46 (d, *J* = 2.2 Hz, 1H), 7.76 (dd, *J* = 9.0, 2.2 Hz, 1H), 7.35 (m, 4H), 7.28 (m, 1H), 7.19 (d, *J* = 9.0 Hz, 1H), 5.82 (d, *J* = 7.9 Hz, 1H), 5.22 – 5.08 (m, 2H), 4.32 (q, *J* = 6.4 Hz, 2H), 3.48 (m, 1H), 80 (m, 2H), 1.65 (m, 2H), 1.25 (m, 1H), 1.50 (m, 1H), 1.41 (m, 3H), 1.36 (t, *J* = 6.4 Hz, 3H). ESI-MS calculated for C_23_H_29_N_2_O_4_: 397.20; found: 397.26 [M+H]^+^.

**4.1.12. Synthesis of compound c4**

Fer-1 (0.1000 g, 0.38 mmol) was dissolved in 5.0 ml of anhydrous THF, then benzyl chloroformate（0.1655g，0.76mmol）was added, and magnetically stirred well. DIPEA (0.3940 g, 1.52 mmol) was added to the solution, protected under N_2_ atmosphere, stirred at room temperature for 15h, and the reaction stopped as monitored by TLC. Added 15mL of ethyl acetate to dilute the reaction solution, wash twice with equal volume of water, and wash once with equal volume of saturated sodium chloride solution. The organic phase is dried with anhydrous Na_2_SO_4_, and concentrated by rotary evaporation to give the crude product. Column chromatography on petroleum ether: ethyl acetate =20:1 gave compound **c4** as a white solid. The yield was 59%. ^1^H NMR (400 MHz, DMSO-d6) δ 8.79 (s, 1H), 8.46 (d, *J* = 2.2 Hz, 1H), 7.76 (dd, *J* = 9.0, 2.2 Hz, 1H), 7.19 (d, *J* = 9.0 Hz, 1H), 5.82 (d, *J* = 7.9 Hz, 1H), 4.32 (q, *J* = 6.4 Hz, 2H), 4.20 (m, 2H), 3.49 (m, 1H), 1.82 (m, 2H), 1.64 (m, 2H), 1.51 (m, 1H), 1.51 – 1.38 (m, 5H), 1.36 (t, *J* = 6.4 Hz, 3H), 1.27 (t, *J* = 6.3 Hz, 3H).; ESI-MS calculated for C_18_H_27_N_2_O_4_: 335.19; found: 335.17 [M+H]^+^.

**4.2. Cell treatment**

HK-2 cells used in the experiments were cultured in a complete medium consisting of DMEM-F12 (50:50) supplemented with 10% fetal bovine serum (FBS), and penicillin-streptomycin (100 IU/mL). All compounds were accurately weighed using an analytical balance with a precision of 0.0001 g. They were dissolved in cell culture-grade DMSO to prepare stock solutions at a final concentration of 10 mM. These stock solutions were stored at 0°C. Compounds that are prone to degradation were freshly prepared before use.

**4.3. In vitro cytotoxicity**

HK-2 cells in the logarithmic growth phase were collected and seeded into 96-well plates at a density of 4,000 cells per well in 100 μL of complete medium. The cells were incubated overnight (approximately 24 hours) in a cell culture incubator at 37°C with 5% CO_2_ to allow for adherence. After cells had adhered, 100 μL of medium containing various concentrations of the compounds (final concentrations: 100 μM, 50 μM, 25 μM, 12.5 μM, 6.25 μM, 3.125 μM) was added to each well. Control and blank groups were also included.

After 48 hours of incubation, 20 μL of an MTT solution in DMSO (5 mg/mL) was added to each well under light-protected conditions. The plates were then incubated for an additional 4 hours under the same conditions. Following this, the medium was discarded, and 200 μL of chromatographic-grade DMSO was added to each well. The absorbance at 570 nm was measured using a microplate reader set to 37°C with 5 minutes of shaking. Cell viability was calculated, and all experiments were performed in triplicate.

**4.4. Anti-Ferroptosis Activity**

HK-2 cells in the logarithmic growth phase were collected and seeded into 96-well plates at a density of 4,000 cells per well in 100 μL of complete medium. After a 24-hour incubation to allow cell adherence, 50 μL of medium containing various concentrations of compounds (final concentrations: 50 μM, 25 μM, 12.5 μM, 6.25 μM, 3.125 μM, 1.563 μM) was added to each well. Control and blank groups were also included. After 2 hours of incubation, 50 μL of medium containing RSL-3 was added to each well. For the control and blank groups, 50 μL of medium without RSL-3 was added. Following 48 hours of incubation, 20 μL of an MTT solution in DMSO (5 mg/mL) was added to each well under light-protected conditions. The plates were incubated for an additional 4 hours. The medium was then discarded, and 200 μL of DMSO was added to each well. Absorbance at 570 nm was measured using a microplate reader set to 37°C with 5 minutes of shaking. Cell viability was calculated, and all experiments were performed in triplicate. The inhibitory effect of the compounds on ferroptosis was assessed by comparing cell viability between the treated groups and the model group. The EC_50_ values for ferroptosis inhibition by the compounds were calculated using GraphPad software.

**4.5. Release of fer-1 in Solution**

The test compounds were dissolved in a solution (PBS:DMSO=9:1). Different equivalents of H_2_O_2_ were added to the solution, and the reaction was carried out at 37°C to simulate the release of the prodrug in vitro. The reaction mixtures were then analyzed using mass spectrometry to confirm the successful release of the prodrug.

**4.6. Anti-apoptotic activity**

HK-2 cells in the logarithmic growth phase were collected, inoculated into a 96-well plate with 4000 cells/well +100 μL complete culture medium, cultured normally for 24 hours, and administered after the cells adhered to the wall. Add the compound solution to be tested, set the final compound concentration to 25 μM, and set the blank group and the control group. After incubation for 2 hours, 50 μL of camptothecin-containing medium was added, and the final concentration gradient of camptothecin was set to: 25 μM, 15 μM, 10 μM, 5 μM, 2.5 μM, and 1.25 μM. After incubation for 24 hours, cell survival rate was measured by MTT method. In the dark, 20 μL of MTT in DMSO solution (5 mg/mL) was added to each well. After incubation under the same conditions for 4 hours, the culture medium was discarded. 200 μL of DMSO was added to each well. The microplate reader conditions were set to 37℃, the plate was vibrated for 5 minutes, and the absorbance at 570 nm was measured with a microplate reader. Cell survival rate was calculated according to the formula, and all experiments were repeated three times. The ability of compounds to inhibit apoptosis was evaluated by the difference in cell survival between the treated group and the apoptosis model group.

**4.7. Anti-necrotic activity**

HK-2 cells in the logarithmic growth phase were collected and seeded into 96-well plates at a density of 4,000 cells per well in 100 μL of complete medium. After a 24-hour incubation to allow for cell adherence, the test compounds were added, with a final concentration of 25 μM for each compound. Blank and control groups were also included. After 2 hours of incubation, 50 μL of medium containing cisplatin was added to each well, with final concentrations of cisplatin set at 5 μg/mL, 2.5 μg/mL, and 1.25 μg/mL. Following 48 hours of incubation, cell viability was measured using the MTT assay. Under light-protected conditions, 20 μL of MTT solution in DMSO (5 mg/mL) was added to each well. The plates were incubated for an additional 4 hours, after which the medium was discarded, and 200 μL of DMSO was added to each well. Absorbance at 570 nm was measured using a microplate reader set to 37°C with 5 minutes of shaking. Cell viability was calculated using the appropriate formula, and all experiments were conducted in triplicate. The ability of the compounds to inhibit cell necrosis was assessed by comparing the cell viability between the treated groups and the necrosis model group.

**4.8. Total Intracellular ROS Level**

HK-2 cells in the logarithmic growth phase were collected and seeded into 96-well plates at a density of 5,000 cells per well in 100 μL of complete medium. After 24 hours of incubation to allow for cell adherence, treatments were administered. The experimental setup included a blank group, a control group treated with 5 μM RSL-3, a group treated with 5 μM RSL-3 and 25 μM fer-1, and groups treated with 5 μM RSL-3 combined with either a1 or b1 at concentrations of 10 μM and 25 μM.

After 2 hours of incubation, 50 μL of medium containing RSL-3 was added to each well. The cells were then incubated for an additional 24 hours. Following incubation, the old medium was removed, and the cells were washed twice with PBS. Under light-protected conditions, 100 μL of a 5 μM DCFH-DA solution (diluted in serum-free medium) was added to each well. The cells were incubated for 30 minutes in the incubator. After incubation, the DCFH-DA solution was removed, and the cells were washed three times with serum-free medium, gently shaking and ensuring complete removal of the solution each time. Finally, 100 μL of basal medium was added to each well, and fluorescence was observed using a high-content imaging system.

**4.9. Lipid ROS level**

HK-2 cells in the logarithmic growth phase were collected, inoculated into a 96-well plate with 5000 cells/well +100 μ L complete culture medium, cultured normally for 24 hours, and administered after the cells adhered to the wall. Set blank group, 5μM RSL-3 control group, RSL-3+25 μM fer-1 group, and RSL-3+**a1**/**b1** group (both concentrations of 10μM and 25 μM were set for **a1**/**b1**). After incubation for 2 hours, add 50 μL of medium containing RSL-3 to each well. Incubate for 24 hours, aspirate and discard the old medium, wash it twice with PBS, add 100 μL of C11-BODIPY(5 μM) solution diluted in serum-free medium to each well in the dark, and continue to incubate in the incubator for 30 minutes. After the incubation for 30 minutes, aspirate and discard the medium, gently shake and suck it clean during washing. Add 100 μL of basic medium to each well, observe fluorescence and take photos with a high content imaging system.

**4.10. ADMET prediction**

Pharmacokinetic predictions of compounds were performed using the ADMET Descriptors module in Discovery Studio software.

**4.11. Statistical analyses**

Statistical analyses were performed using the GraphPad Prism 9 software (GraphPad Software, CA). Data were collected from 3 independent biological repeats and expressed as means ± SD.


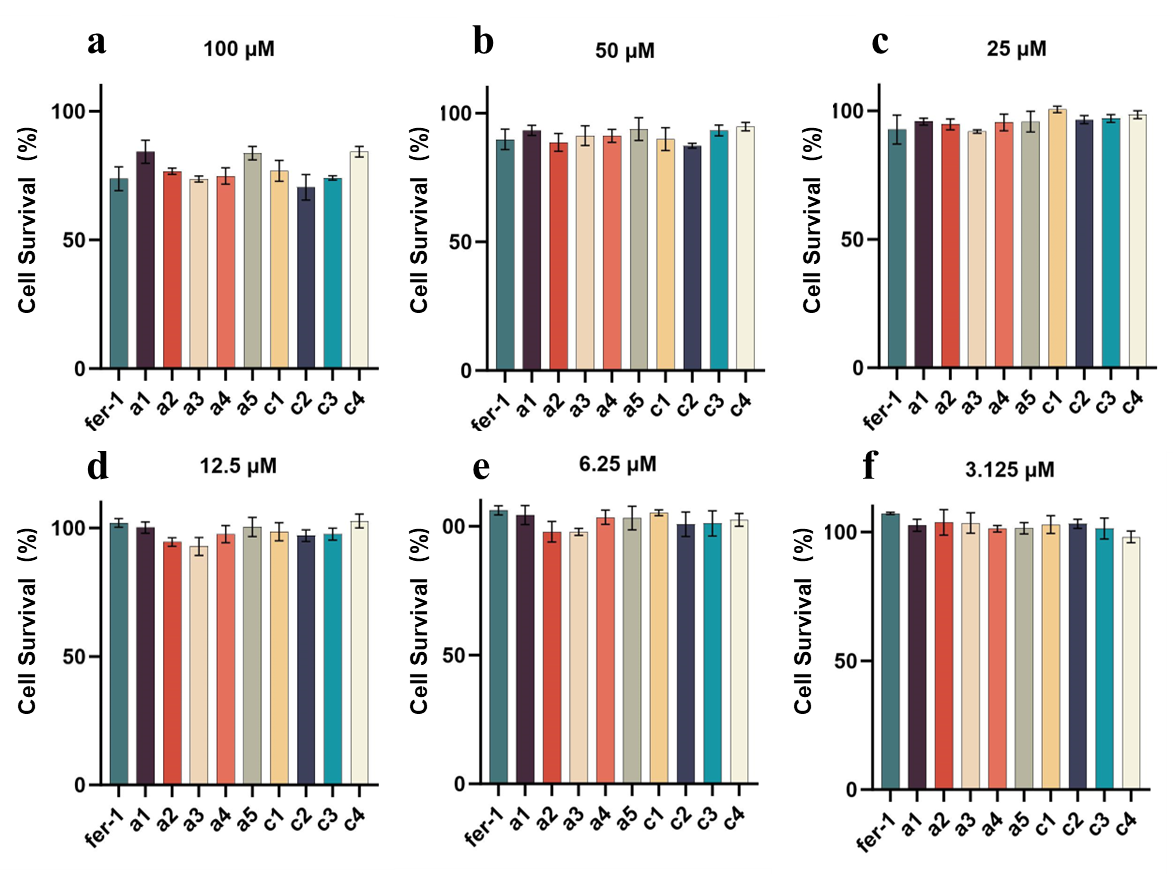


Figure S3. Effects of compounds on cell survival. Compound concentrations are as follows: (a)100μM、(b)50μM、(c)25μM、(d)12.5μM、(e)6.25μM、(f)3.125μM. p < 0.01 compared with control group


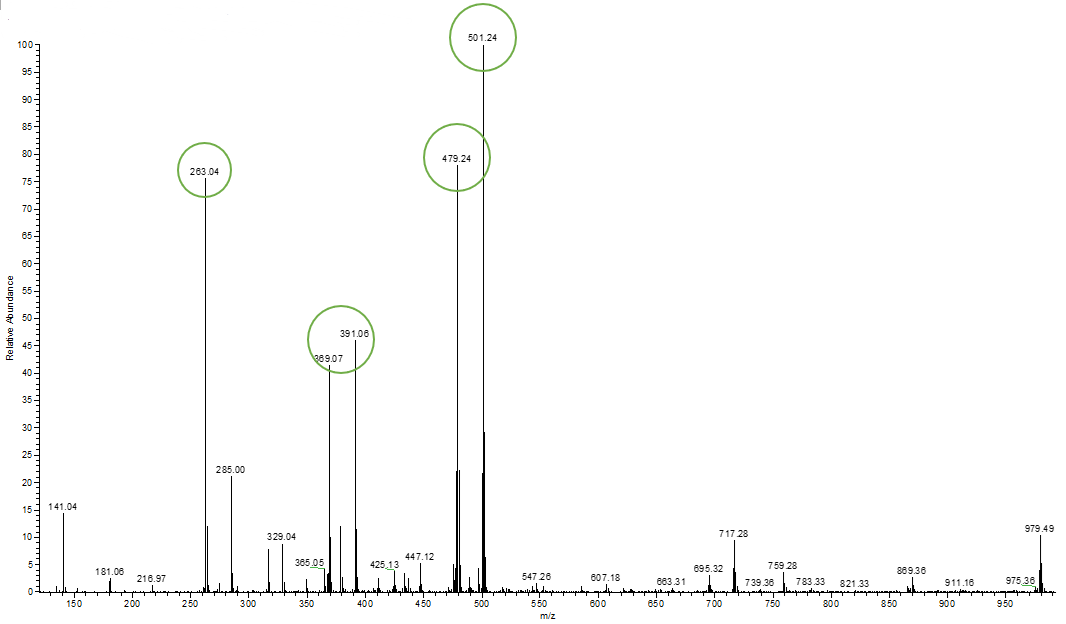

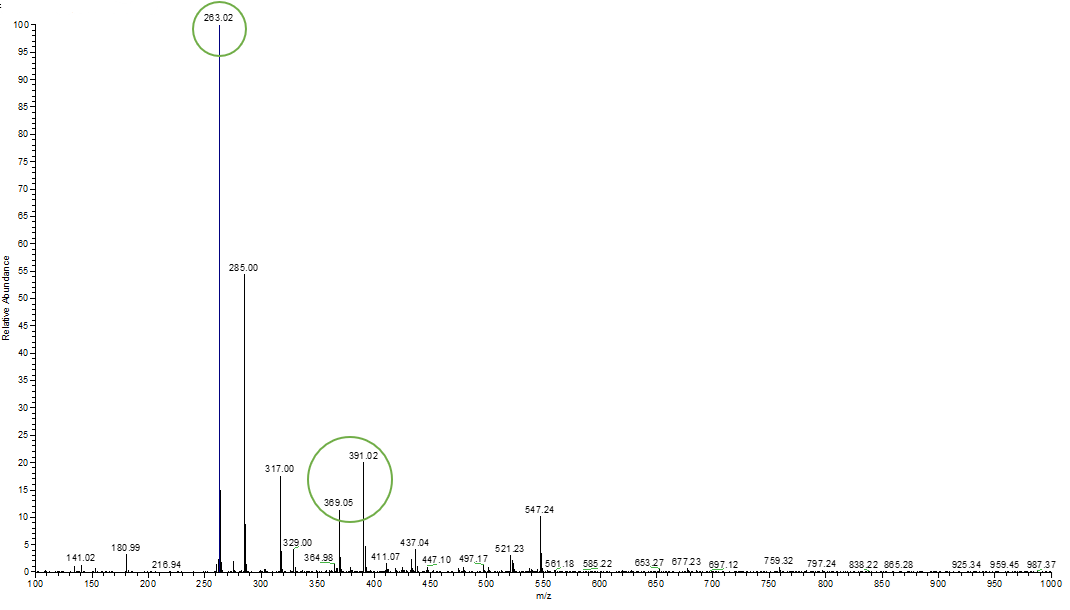


263.04

369.07

479.24

501.24

263.02

369.05

Figure S4. Mass spectrum of a1 after adding H_2_O_2_
